# Differences in the genomic profiles of cell‐free DNA between plasma, sputum, urine, and tumor tissue in advanced NSCLC

**DOI:** 10.1002/cam4.1935

**Published:** 2019-02-14

**Authors:** Zhen Wu, Zhen Yang, Chun Sun Li, Wei Zhao, Zhi Xin Liang, Yu Dai, Qiang Zhu, Kai Ling Miao, Dong Hua Cui, Liang An Chen

**Affiliations:** ^1^ Respiratory Department of Chinese PLA General Hospital Beijing China

**Keywords:** cell‐free DNA, liquid biopsy, lung cancer, next‐generation sequencing, sputum

## Abstract

Liquid biopsy has provided an efficient way for detection of gene alterations in advanced non‐small‐cell lung cancer (NSCLC). However, the correlation between systematic determination of somatic genomic alterations in liquid biopsy and tumor biopsy still remained unclear, and the concordance rate between cell‐free DNA (cfDNA) and matched tumor tissue DNA needs to be increased. A prospective study was performed to detect differences in genetic profiles of cfDNA in sputum, plasma, urine, and tumor tissue from 50 advanced NSCLC patients in parallel by the same next‐generation sequencing (NGS) platform. Driver genes alterations were identified in cfDNA sample and matched tumor sample, with an overall concordance rate of 86% in plasma cfDNA, 74% in sputum cfDNA, 70% in urine cfDNA, and 90% in cfDNA of combination of plasma, sputum, and urine. And the concordant rate of cfDNA in sputum in patients with smoking history was higher than that in patients without history of smoking (89% vs. 66%, *P* = 0.033) and equal to that in plasma cfDNA of the smoking patients (89% vs. 89%). In conclusion, sputum cfDNA can be considered as an alternative medium to liquid biopsy, while the complementarity of genomic profiles in cfDNA among plasma, sputum, and urine was beneficial to detect more diver genes alterations and improve the utility of liquid biopsy in advanced NSCLC (Liquid Biopsy for Detection of Driver Mutation in NSCLC; NCT02778854).

## 1 INTRODUCTION

With the development of molecular‐targeted therapy in non‐small cell lung cancer (NSCLC), the outcomes of advanced NSCLC have significantly improved^1^, ^2^ for both newly diagnosed patients and acquired drug‐resistant patients. The identification of genotype based on tumor tissue has become the initial procedure before selecting personalized treatment for advanced NSCLC patients. Epidermal growth factor receptor (*EGFR*) mutations, amplification of MET, anaplastic lymphoma kinase (*ALK*), *ROS1, *and *RET *fusions are the representative driver oncogene of advanced NSCLC.^4^

However, many patients have no access to tissue biopsy because of the invasiveness of the procedure and intolerance of the operation; thus, these patients have no chance of receiving targeted therapy before the plasma was recommended for genetic detection.^5^, ^6^ Especially for acquired drug‐resistant patients after receiving first‐line EGFR‐TKI, worse health conditions make it more difficult to accept rebiopsy for guiding personalized therapy.^7^

Liquid biopsy, characterized by noninvasiveness, easy accessibility, and good repeatability, is increasingly used for gene mutation detection in NSCLC^8^, ^9^ and drug resistance monitoring after the first‐line targeted therapy^10^, ^11^ due to cell‐free DNA(cfDNA) released into circulation through apoptosis or necrosis by cancer cells.^13^, ^14^ However, the relationships between liquid samples including plasma and urine and tumor tissue have yet not clarified clearly due to the tumor heterogeneity and the restriction of the testing method.^15^, ^16^ Actually, there is a large amount of cfDNA in the sputum of patients with NSCLC, and the value of this material for testing genetic mutations in NSCLC needs further exploration.^18^ Therefore, we plan to combine plasma, sputum, and urine to further explore the concordance and relationship between these liquid samples and tumor tissues. The detection method is another issue needed to be considered carefully since sensitivities of different methods are diverse.^8^, ^19^, ^20^ NGS, as a sensitive method, can discover a tremendous number of driver gene variations involving therapeutic target genes and acquired drug‐resistant genes simultaneously and has been increasingly applied to clinical practice recently.^22^, ^23^ Therefore, NGS was chosen to conduct our research.

With the recently proposed concept of co‐occurring genetic alterations, the influence of concurrent gene mutations on the prognosis of lung cancer has received increasing attention.^24^, ^25^ Investigation of differences in the gene variation spectrum between body fluid samples and tissue samples, which involves the detection of multiple genes, has become the major focus. Therefore, to further clarify the differences in genomic profiles between different body fluid samples and tissue samples and increase the utility of liquid biopsy in clinical practice, we conducted a prospective study to detect multigenes in multiliquid samples, including sputum, plasma, and urine, and tumor tissue in parallel by NGS 400‐gene panel under the same detection platform.

## 2 MATERIALS AND METHODS

### 2.1 Patients

From October 2015 to December 2017, 50 patients with advanced NSCLC admitted to the Respiratory Department of Chinese People's Liberation Army General Hospital were enrolled in the study (NCT02778854). All patients participating in this study were required to meet the criteria: (a) The patients must be diagnosed with stage IIIB or IV NSCLC (Table 1). (b) All patients accepted biopsy with sufficient tumor tissue to detect genetic mutations. (c) The patients must provide sufficient plasma, urine, and sputum to finish the gene detection. Finally, 50 patients meeting the criteria were recruited, while 60% of patients were female and 70% of patients were nonsmoking because female patients are more likely to follow the instruction to provide the required liquid samples including plasma, urine, and sputum, and all the female patients and 25% of male patients enrolled in our research did not smoke. The study was approved by the hospital's ethics committee and signed informed consent was provided by patients or their families.

**Table 1:** Clinical and demographic characteristics of patients

	All patients (n = 50)
Age, median (range)	61 (36‐81)
Sex, N (%)	
Male	20 (40)
Female	30 (60)
Smoking, N (%)	
Yes	15 (30)
No	35 (70)
Lines of therapy	
0	32 (64)
1	18 (36)
Histology, N (%)	
Adenocarcinoma	48 (96)
Squamous	1 (2)
NOS	1 (2)
Disease stages, N (%)	
IIIb	7 (14)
IV	43 (86)
Metastatic stages	
M1a	7 (14)
M1b	8 (16)
M1c	28 (56)
Metastatic site	
Intrathoracic metastasis	7 (14)
Extrathoracic metastasis	36 (72)
Number of metastatic organs	
1	10 (20)
>1	26 (52)

### 2.2 Sample collection and processing

Paired body fluids, including sputum, plasma, and urine, of the same patient were collected prior to the first line of therapy in newly diagnosed patients or before changing the treatment regimen in patients with acquired drug resistance. Nearly 5 mL sputum was collected in mixed solution with an equal volume of Saccomanno's fixative and 0.005% dithiothreitol solution at a 1:1 ratio.^26^ The mixture was filtered through a nylon membrane after a 10‐minute hot water bath at 37°C and centrifuged at 268 *g* for 10 minutes to separate the supernatant from the cell pellet. The supernatant was then further centrifuged at 14 000 *g* for 10 minutes at 4°C to remove any remaining cell debris. The specimens were considered adequate only if alveolar macrophages and bronchial epithelial cells were present in hematoxylin‐eosin staining of the cell pellet.^18^ Approximately 10 mL peripheral venous blood was collected in a standard ethylenediaminetetraacetic acid (EDTA) tube, and the follow‐up procedures were performed according to the related procedures of previous studies.^21^ Almost 15‐30 mL middle urine of the same patient was collected in the morning and mixed with EDTA (0.5 mol/L, pH 8.0) to reach a final concentration of 10 mmol/L EDTA. The mixture was then centrifuged at 268 *g* to separate the supernatant. Finally, the final supernatants of these three liquids were stored at −80°C. All above‐mentioned procedures were finished within 2 hours of sample collection. And cfDNA was purified from all of these liquid samples (2 mL plasma, 2 mL sputum, and 6‐10 mL urine) by a QIAamp Circulating Nucleic Acid kit (Qiagen, Duesseldorf, Germany), and DNA from peripheral blood lymphocytes (PBLs) was extracted by a Gentra Puregene Blood kit (Qiagen). Fresh tumor tissues (no <1 cm, 1‐3 strips) were obtained by tumor biopsy. At least 10% of the tumor cells found in the sections of fresh tumor tissue were considered qualified specimens, and DNA was subsequently extracted and analyzed with NGS (HiSeq platform; Illumina, San Diego, CA). DNA was quantified by a Nanodrop 2000 (Thermo Fisher Scientific, Waltham, MA) and Qubit 3.0 using a dsDNA HS Assay kit (Life Technologies, Waltham, MA) according to the manufacturer's recommendations. All of these extraction and analyzing procedures were processed at a CAP/CLIA‐certified clinical diagnostic laboratory.

### 2.3 Library preparation and NGS

Sequencing libraries were prepared using a KAPA Hyper Prep kit (KAPA Biosystems, Boston, MA) with an optimized manufacturer's protocol for different samples types (plasma, sputum, and urine shared the same protocol). In brief, 250 ng‐1 µg genomic DNA fragments or 10‐250 ng cfDNA underwent end‐repairing. A‐tailing and ligation with indexed adapters sequentially, followed by size selection of genomic DNA using Agencourt AMPure XP beads (Beckman Coulter, Pasadena, CA). Finally, libraries were amplified by PCR and purified for target enrichment. Hybridization‐based target enrichment was performed using GeneseeqOne™ 416‐gene panel (Nanjing Geneseeq Technology Inc., Nanjing, China). Library fragment size was determined by an Agilent Technologies (Palo Alto, CA) 2100 Bioanalyzer. The target‐enriched library was then sequenced on HiSeq4000 NGS platforms (Illumina). The following analysis was performed according to previous research using the same NGS platform and panel.^27^

Human genome (hg19) was applied as reference to map the reads. Base quality score recalibration and local realignment around the indels were used with the Genome Analysis Toolkit (GATK 3.4.0; https://software.broadinstitute.org/gatk/), which was also used to detect germline mutations. VarScan2 (23) was applied for somatic mutation detection. Somatic variant calls with at least 0.1% mutant allele frequency (MAF) and with at least 3 supporting reads were retained. Common SNPs were filtered out by dbSNP (v137) and the 1000 genomes database, followed by annotation applied with ANNOVAR. ADTEx (http://adtex.sourceforge.net) with default parameters were performed to detect copy number variations (CNVs). Somatic CNVs were determined by applying the paired normal/tumor samples to meet the standard that each exon with the cut‐off that 0.65 for copy number loss and 2.00 for copy number gain.

### 2.4 Statistical analysis

Results of genetic testing of tumor tissue are considered the reference for comparison with that of cfDNA in plasma, sputum, and urine. The same mutations detected in both matched tumor and cfDNA samples were classified as true positives; true negatives were identified as those where both matched tumor and cfDNA samples had no mutations; mutations identified in cfDNA which were not found in tumor tissue DNA were classified as false positives; and mutations identified in tumor tissue DNA but not in cfDNA were classified as false negatives. Concordance rate was defined as the ratio of the sum of the number of true positive and true negative to total enrolled patients. The sensitivity and specificity of individual sample or unique gene alterations were compared with tissue biopsy by the chi‐square test. All statistical tests were bidirectional, and differences were considered significant when *P* < 0.05. Statistical analysis was performed by the SPSS 22 program (SPSS Inc., Chicago, IL).

## 3 RESULTS

### 3.1 Quality control of the sequencing results

**Figure 1:**
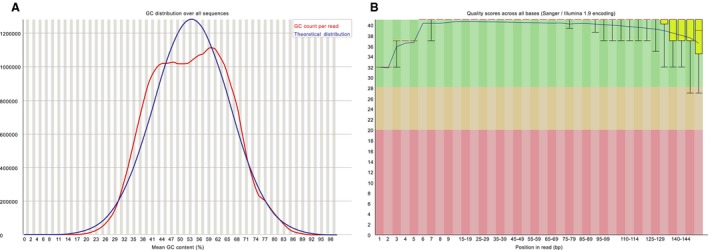
Quality control of sequencing with Hiseq4000. A, The GC distribution over all sequences of cfDNA in liquid samples. B, Quality scores across all bases of cfDNA in liquid samples

The GC content of all bases is approximately 50% (Figure 1A). QC scores for all bases, which indicated the accuracy of the sequencing results, are presented in Figure 1B. Majority of the length of the cfDNA fragment of plasma and sputum samples were about 160 bp, while which of urine was just about 100 bp. The sequencing depth was more than 100× mean coverage for PBLs and more than 800× mean coverage for tumor tissue samples both by non‐PCR duplicate read pairs. While in cfDNA, the sequencing depth was more than 3000× after removing PCR duplicates in spite of variable depth obtained for analysis.

### 3.2 Distribution of tumor‐associated driving genes mutation in cfDNA from different body fluid samples and tumor DNA from tissue

**Figure 2:**
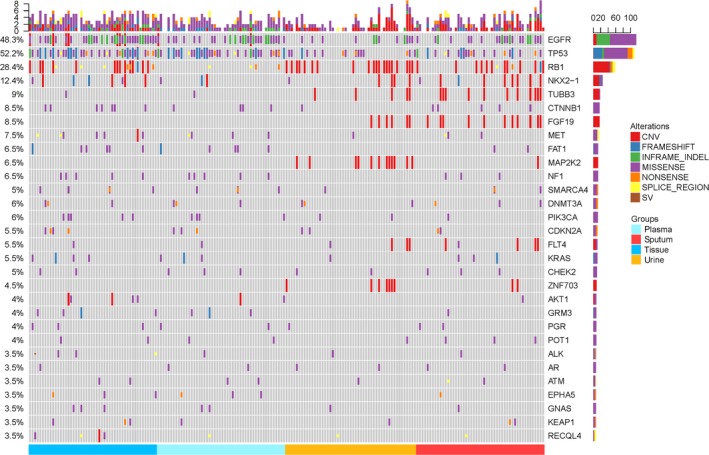
Characteristics of top 30 in most frequently mutated genes of the whole overall genomic profiles in cfDNA of tissue, plasma, urine, and sputum

**Figure 3:**
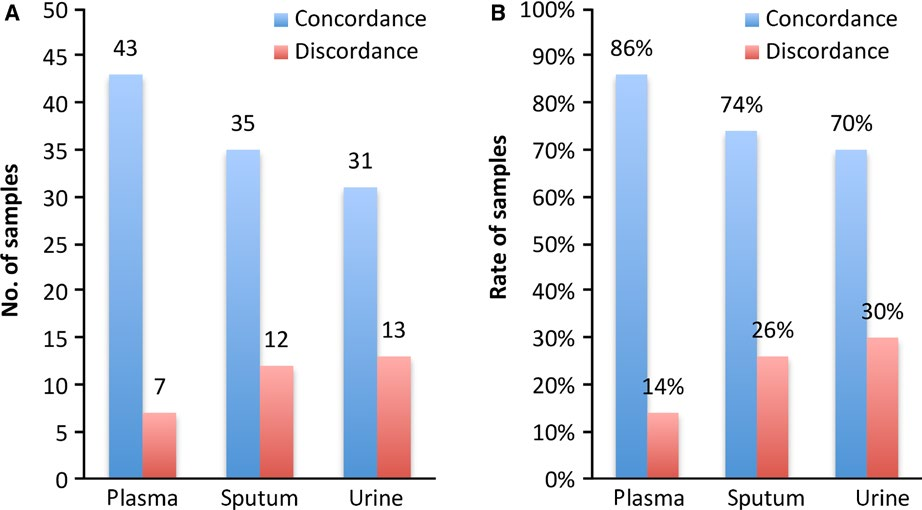
Concordances of tumor tissue DNA and matched cfDNA in plasma, sputum, and urine samples. A, The number of patients with concordant and discordant mutations identified in cfDNA samples and matched tumor tissue DNA. B, The concordant rate of concordant mutations and discordant mutations identified in matched tumor tissue DNA and cfDNA samples

Except that three sputum samples and six urine samples from nine different patients (all from the group of newly diagnosed patients) failed to sequence due to a lack of cfDNA quality or quantity, all of the other samples finished the test. The average number of mutations in the driver genes was 8.20 ± 4.030 (410/50) in the tissue, 6.08 ± 3.72 (292/48) in the plasma, 5.50 ± 4.009 (242/44) in the sputum, and 4.02 ± 2.30 (257/39) in the urine, and the difference between the groups was statistically significant (*P* < 0.001). The results of mean allele frequency of driving gene mutations in each type of sample were presented in the following order: tissue (15.87%) > plasma (5.72%) > sputum (3.45%) > urine (2.91%), *P* < 0.001. The characteristics of top 30 in the most frequently mutated genes among different groups and different types of samples were shown in Figure 2. The concordant mutations identified in both cfDNA and tumor tissue tDNA were in 42 of 48 samples (88%) in plasma, 76% (34/45) in sputum, and 69% (29/42) in urine, while the overall concordance rate (including matched mutations and matched nonmutations) of cfDNA to matched tumor tissue DNA was 86% (43/50 samples) in plasma, 74% (35/47 samples) in sputum, and 70% (31/44) in urine (Figure 3). Taken all detection results of plasma, sputum, and urine into consideration, the overall sensitivity of combination of three kinds of liquid samples was 92% (44/48), which was more than that of combination of sputum and urine—75% (36/48), plasma and urine—90% (43/48), or plasma and sputum—90% (43/48). Although the difference was not statistically significant (*P* = 0.063), it presented a positive tendency towards the value of combining multiple biologic liquids for liquid biopsy. While overall concordant rate of combination of 3 kinds of liquid samples was 90% (45/50), which was more than that of combination of sputum and urine—78% (39/50), plasma and urine—88% (44/50), or plasma and sputum—88% (44/50), the difference did not reach the statistically significant level (*P* = 0.301).

Patients characteristics potentially associated with overall concordant rate of cfDNA to matched tumor tissue DNA were analyzed in each subgroup. Of the characteristics listed in Table 1, the overall concordance in cfDNA of plasma in patients with extrathoracic metastasis was higher than in patients with intrathoracic metastasis (92% vs. 57%, *P* = 0.045; Table 2). And the history of smoking was considered to be associated with overall concordant rate of sputum cfDNA to matched tumor tissue DNA significantly, while overall concordance between sputum cfDNA and tDNA was higher in patients with history of smoking (89%) vs. without history of smoking (66%), and this difference was statistically significant (*P* = 0.033; Table 2). Moreover, the concordant rate of cfDNA in sputum was equal to that of plasma cfDNA in patients with history of smoking (89% vs. 89%).

**Table 2:** Correlation between overall concordance of cfDNA samples to matched tumor tissue DNA and clinical characteristics

	Plasma (%)	Sputum (%)	Urine (%)	Total (%)
Sex				
Male	95 (19/20)	89 (17/19)	79 (15/19)	95 (19/20)
Female	80 (24/30)	64 (18/28)	64 (16/25)	87 (26/30)
*P*	0.279	0.109	0.282	0.336
Smoking				
Yes	95 (18/19)	89 (17/18)	78 (14/18)	95 (18/19)
No	81 (25/31)	66 (18/29)	65 (17/26)	87 (27/31)
*P*	0.330	0.033	0.376	0.362
Metastatic site				
Intrathoracic	57 (4/7)	57 (4/7)	57 (4/7)	71 (5/7)
Extrathoracic	92 (33/36)	82 (28/34)	76 (25/33)	92 (33/36)
*P*	0.045	0.165	0.369	0.045
No. of metastatic sites				
1	80 (8/10)	63 (5/8)	50 (4/8)	80 (8/10)
>1	96 (25/26)	88 (23/26)	84 (21/25)	96 (25/26)
*P*	0.181	0.126	0.074	0.181

### 3.3 Driver genes mutations detection in matched tumor tissue DNA and cfDNA samples

**Figure 4:**
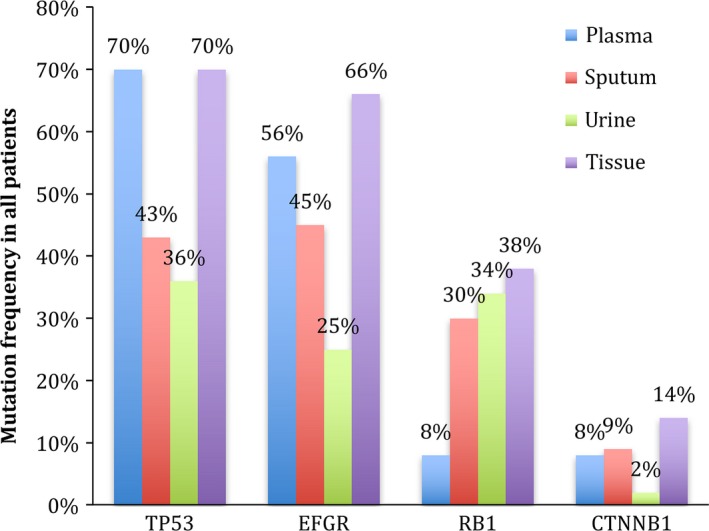
Comparisons of mutation rates of TP53, EGFR, RB1, and CTNNB1 in different types of samples

The genes with the highest mutation frequency were TP53 and EGFR, which accounted for 52.2% and 48.3% in the samples successfully sequenced, respectively (Figure 2). Interestingly, among the top 4 of most frequently mutated genes in all types of samples, rates of EGFR and TP53 mutations ranked top 2 among the cfDNA from any kinds of samples and tumor tissue DNA, while the frequency of RB1 mutation was the third ranking in cfDNA from urine (34%), sputum (30%), and tumor tissue (38%), however, which in plasma cfDNA (8%) was far less than that in tumor tissue and the rest of cfDNA (*P* = 0.003; Figure 4). It was determined that the top 10 of the most frequently driver mutations in different types of samples were distinctive (Table 3A). The consistent rates of top 10 driver mutations were 90% in plasma, 70% in sputum, and 60% in urine when compared with tumor tissue, and which increased to 100% after combining the plasma, sputum, and urine to finish liquid biopsy at the same time. And the top 5 of the most frequently copy number variations (CNV) were also different in various liquid samples. The consistent rates of these CNVs were 100% in comparison with tumor tissue after combination of these 3 kinds of liquid samples (Table 3B).

**Table 3:** (A) The top 10 in the most frequently driver mutations in plasma, sputum, urine, and tumor tissue. (B) The top 5 in the most frequently copy number variations in plasma, sputum, urine, and tumor tissue

Rank	Driver genes (n, %)			
	Plasma	Sputum	Urine	Tumor tissue
A				
1	TP53 (34, 68%)	TP53 (20, 43%)	TP53 (13, 30%)	TP53 (35, 70%)
2	EGFR (28, 56%)	EGFR (20, 43%)	EGFR (10, 23%)	EGFR (33, 66%)
3	NF1 (5, 10%)	CTNNB1 (4, 9%)	DNMT3A (3, 7%)	CTNNB1 (7, 14%)
4	FAT1 (5, 10%)	NF1 (3, 6%)	PIK3CA (2, 5%)	RB1 (6, 12%)
5	PIK3CA (4, 8%)	MET (3, 6%)	CHEK2 (2, 5%)	MET (6, 12%)
6	MET (4, 8%)	SMARCA4 (3, 6%)	CDKN2A (2, 5%)	FAT1 (6, 12%)
7	CTNNB1 (4, 8%)	KRAS (3, 6%)	AR (2, 5%)	PIK3CA (5, 10%)
8	RB1 (4, 8%)	DNMT3A (3, 6%)	AMER1 (2, 5%)	NF1 (5, 10%)
9	KRAS (3, 6%)	CHEK2 (3, 6%)	CTNNB1 (1, 2%)	KRAS (4, 8%)
10	CHEK2 (3, 6%)	POT1 (3, 6%)	KRAS (1, 2%)	DNMT3A (4, 8%)
B				
1	EGFR (2, 4%)	RB1 (14, 30%)	RB1 (12, 27%)	RB1 (13, 26%)
2	MYCN (2, 4%)	ZNF703 (6, 13%)	NKX2‐1 (7, 16%)	EGFR (6, 12%)
3	MYC (1,2%)	FGF19 (5, 11%)	FGF19 (5, 14%)	MYC (5, 10%)
4	NKX2‐1 (1, 2%)	NKX2‐1(4, 9%)	TUBB3 (5, 14%)	NKX2‐1 (4, 8%)
5	AKT1 (1, 2%)	TUBB3 (3, 6%)	ZNF703 (2, 5%)	AKT1 (2, 4%)

TP53 mutations were the leading driver gene detected in plasma, accounting for 70% of all the patients. Of the 42 matched samples with concordant alterations in tumor tissue DNA and plasma cfDNA, alterations were found as follows: TP53 (74%), EGFR (62%), PIK3CA (10%)，and KRAS (7%). Of the seven matched samples with discordant results, six had unpaired mutations identified in tumor tissue DNA but not in plasma cfDNA in the following genes: TP53 (67%), EGFR (33%), and RB1 (33%); and 2 of 7 paired samples had mutation in plasma cfDNA but not in tumor tissue DNA in TP53 (100%), ERBB2 (50%), and EGFR (50%).

EGFR was the most frequently mutated (45%) driver genes in sputum. Of the 34 matched samples with concordant alterations in tumor tissue DNA and sputum cfDNA, alterations were found as follows: EGFR (44%), TP53 (53%), CTNNB1 (12%), and RB1 (12%). Of the 12 matched samples with discordant results, 11 had unmatched mutation detected in tumor tissue DNA but not in sputum cfDNA: EGFR (55%), TP53 (67%), RB1 (36%), while one sample had EGFR mutations in sputum cfDNA but not in tumor tissue DNA.

TP53 mutations were the leading driver gene detected in urine (36%). Of the 29 matched samples with concordant alterations in tumor tissue DNA and urine cfDNA, mutations were detected in the following driver gene mutations: EGFR (34%), TP53 (45%), RB1 (30%), and DNMT3A (7%). Of the 13 matched samples with discordant samples, 11 had unmatched mutations identified in tumor tissue DNA but not in urine cfDNA: EGFR (73%), TP53 (91%), RB1 (18%), and CTNNB1 (18%). Two sample had TP53 mutation in urine cfDNA but not in tumor tissue DNA. There were only five matched liquid cfDNA and tumor tissue DNA samples with discordant results after combining the plasma, sputum, and urine, which was less than combination before. Of these five matched samples, EGFR mutations were identified in three tumor tissue while ERBB2 and TP53 mutations were identified in cfDNA samples of each patient, respectively.

## 4 DISCUSSION

To our knowledge, this prospective research is the first one to conduct an integrated liquid biopsy based on three types of liquid samples including plasma, sputum, and urine compared with tumor tissue under the same NGS platform in advanced NSCLC, aiming to explore the differences in genomic profiles among different types of samples and evaluate the value of combining multisamples in liquid biopsy to improve its clinical application in NSCLC. The study confirmed that both differences and similarities existed in genomic profiles between different body fluid samples and tissues under the same detection platform. The order of whether the average number of driven gene mutations and the mean allele frequency of driving genes alterations in individual type of sample was same: tissue > plasma > sputum > urine. The overall concordant rates of cfDNA to matched tumor tissue DNA were 86% (43/50) in plasma, 74% (35/47) in sputum, 70% (31/44) in urine, and 90% (45/50) in combination of these three kinds of liquid samples. Also, with the combination of plasma, sputum, and urine, the overall concordant rate of cfDNA to matched tumor DNA increased more than combination of any other two kinds of samples, which means more driver gene alterations might be found by combination of plasma, sputum, and urine in liquid biopsy.

Of the discordant mutation results in matched cfDNA sample and tumor tissue sample, extra EGFR and ERBB2 mutations can be found in plasma cfDNA and sputum cfDNA but not in tumor tissue DNA and extra TP53 mutation can be detected in urine cfDNA but not in tumor DNA. Studies in plasma and urine have already shown that urine cfDNA and plasma cfDNA can find extra T790M that was not detected in tumor tissue.^28^ Because of the existence of tumor heterogeneity, it may be impossible that single tissue biopsy especially the small sample biopsy can cover the overall genomic profile of the entire tumor, while the cfDNA in liquid samples released from the circulating tumor cells may represent the genomics not only from primary tumor site but also from metastasis sites.^29^, ^30^ Consistent with previous studies, different liquid samples including plasma, sputum, and urine have their own unique genomic profile distinctive from tumor tissue and combination of plasma, sputum, and urine can detect more driver genes mutations including EGFR, ERBB2, and others to guide the clinical practice.

**Figure 5:**
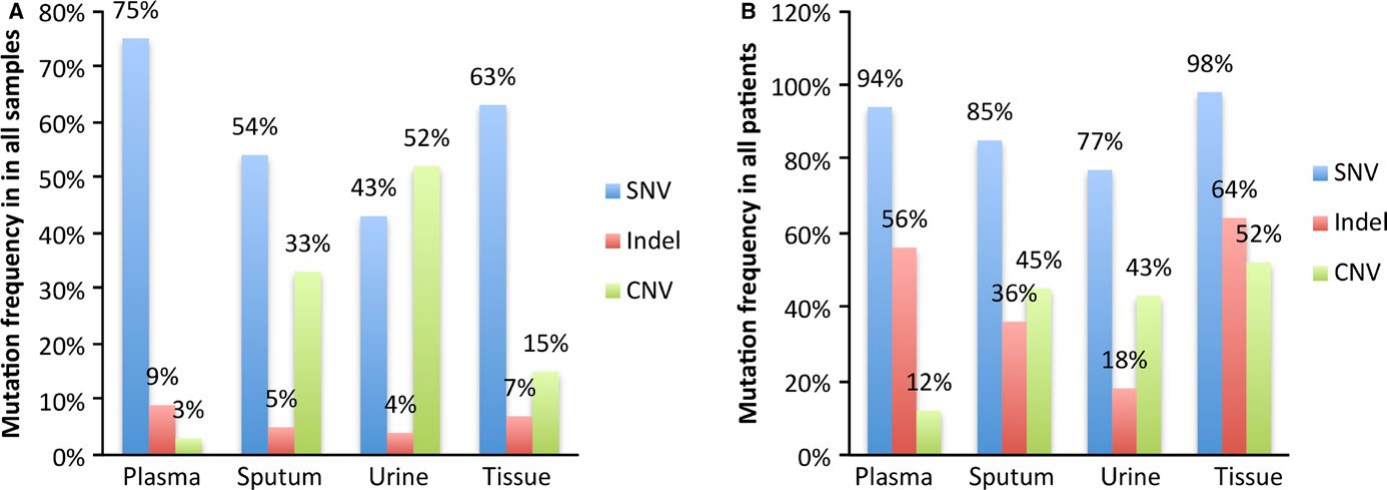
A, The distributions of types of gene variations in different liquid samples. B, The patients with different types of gene variations detected by different liquid samples

As reported in previous researches, EGFR and TP53 were still the driver genes with the highest mutation frequency (48.3% and 52.2%) in any types of samples.^31^ And the rates of EGFR and TP53 alterations were higher in plasma than in sputum and urine (Figure 4), while RB1 aberrations, as the third ranking in most frequently mutated genes, tended to be detected more often in tissue (38%), urine (34%), and sputum (30%) than in plasma (8%). RB1 have been shown to be associated with many kinds of cancer and demonstrated to be a common driver gene in lung cancer.^24^, ^32^, ^33^ Moreover, 38% patients were diagnosed with RB1 aberrations by tumor biopsy, which means the results of RB1 in urine and sputum were consistent with that of tumor tissue, and it is neither false operation nor contamination by genomic profiles of normal tissue that more RB1 aberrations can be found by urine and sputum than plasma. Mutations of RB1 in samples detected were mainly CNVs, although the most common type of genes alterations was single nucleotide variations (SNV; 75%) in plasma, SNV (54%) in sputum, and CNV (52%) in urine (Figure 5A), the patients diagnosed with SNVs were still the majority of all detected by any kind of sample and the percentage of patients with CNVs were similar in sputum (45%), urine (43%), and tumor tissue (52%), *P* = 0.651 (Figure 5B). As the previous study demonstrated that the genomic profiles were distinctive in different liquid samples, multiple CNVs were mainly identified in CSF cfDNA rather than in plasma cfDNA, and driver genes in cfDNA in pleural effusions and ascites were also different from that in plasma,^34^ while our study also demonstrated that the genomic profiles in urine and sputum were distinct with that in plasma since more CNVs can be detected in urine and sputum than in plasma but it was consistent with tumor tissue, which was exactly the tumor heterogeneity reflected in different liquid samples. Moreover, the top 10 of the most frequently driver gene mutations and top 5 of the most frequently CNVs were also distinctive in different kinds of samples, but the overall consistent rate can elevate to 100% after combining these three kinds of liquid samples. Therefore, it is necessary to combine different samples to increase the utility of liquid biopsy due to the difference and complementary genomic profiles in each individual type of sample.

Moreover, it was the first time that sputum cfDNA was identified as a valuable sample for liquid biopsy. Although the concordant rate of cfDNA in sputum to matched tumor DNA was 74%, extra driver genes mutations including EGFR and TP53 can be found in sputum cfDNA but not in tissue and plasma. The patients with a history of smoking had a higher concordant rate of cfDNA in sputum than patients without a history of smoking (89% vs. 66%, *P* = 0.033). Previous research has found that total amount of cfDNA from smoking patients was relatively more than that from never smoking patients,^18^ which may lead to more cfDNA to be released from cancer cells and enable more driver genes mutations to be detected in sputum cfDNA. And the concordant rate of cfDNA in sputum was equal to that in plasma cfDNA for smoking people (89% vs. 89%). As a result, these indicate for the first time that either sputum or plasma driver genes testing may be identified as an alternative to tumor tissue biopsy for smoking patients. Sputum can be the preferable option since it represents a truly noninvasive alternative which can be collected by patients themselves at their own home, and both diagnostic and dynamic monitoring detection can be made available without visits to the hospital.

Of course, our study has some limitations. Firstly, larger sample size is needed in general to validate our conclusions. Moreover, some results, like CNVs, were seemed to be more likely to be found in urine and sputum, needed to be further validated, and explored in the future research. In the follow‐up study, we will further validate our present results and improve the storage and processing conditions for sputum and urine to increase the sensitivity of mutation detection.

In conclusion, this study confirmed the clinical value of sputum cfDNA for detecting driven genes alterations in NSCLC, and sputum can be considered as an alternative to tumor biopsy in advanced NSCLC especially for smoking patients. It was also illustrated that the difference of genomic profiles between different liquid samples enabled the combination of various liquid samples including sputum, plasma, and urine to improve the clinical utility of liquid biopsy. As a result, it might allow more patients with advanced NSCLC to undergo comprehensive liquid biopsy when tissue biopsy is not available for personalized and accurate treatment.

## CONFLICT OF INTEREST

The authors have no conflict of interest.
